# A Statistical Analysis of Fluid Interface Fluctuations: Exploring the Role of Viscosity Ratio

**DOI:** 10.3390/e26090774

**Published:** 2024-09-10

**Authors:** Selwin Heijkoop, David Rieder, Marcel Moura, Maja Rücker, Catherine Spurin

**Affiliations:** 1 Mechanical Engineering Department, Eindhoven University of Technology, 5612 AZ Eindhoven, The Netherlands; 2 Eindhoven Institute of Renewable Energy Systems, 5612 AZ Eindhoven, The Netherlands; 3 PoreLab, The Njord Centre, Department of Physics, University of Oslo, 0316 Oslo, Norway; 4 Max Planck Institute for Polymer Research, 55128 Mainz, Germany; 5 Energy Science & Engineering, Stanford University, Palo Alto, CA 94305, USA

**Keywords:** multiphase flow, relative permeability, CO_2_ storage, fluid flow, carbon sequestration

## Abstract

Understanding multiphase flow through porous media is integral to geologic carbon storage or hydrogen storage. The current modelling framework assumes each fluid present in the subsurface flows in its own continuously connected pathway. The restriction in flow caused by the presence of another fluid is modelled using relative permeability functions. However, dynamic fluid interfaces have been observed in experimental data, and these are not accounted for in relative permeability functions. In this work, we explore the occurrence of fluid fluctuations in the context of sizes, locations, and frequencies by altering the viscosity ratio for two-phase flow. We see that the fluctuations alter the connectivity of the fluid phases, which, in turn, influences the relative permeability of the fluid phases present.

## 1. Introduction

Understanding multiphase flow through porous media is integral across many fields and plays a critical role in energy applications such as geologic carbon storage or hydrogen storage [1,2,3]. The movement of fluids in the subsurface is modelled using relative permeability functions [4,5]. In this, it is assumed that each fluid present exists in its own static, connected pathway. All regions of a fluid not connected across the pore space are assumed to be trapped [6,7].

**Figure 1:**
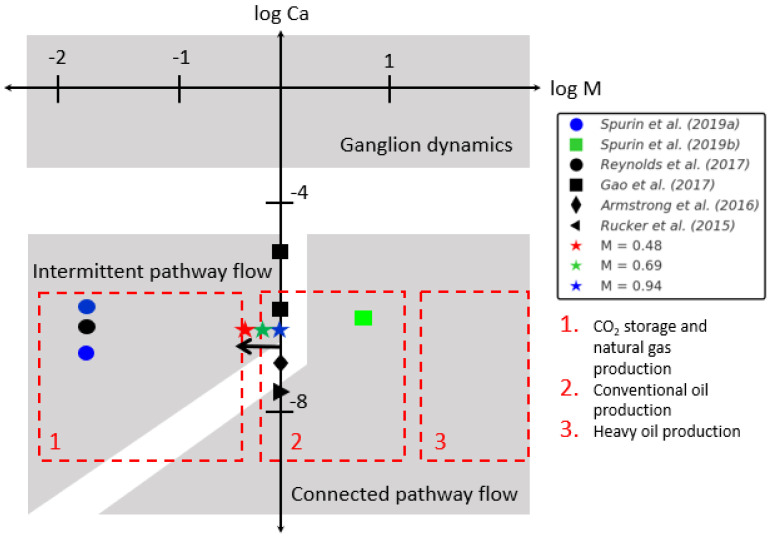
Phase diagram for steady-state two-phase flow experiments [8,10,13,15,19,20]. The white areas denote the transition zones between the different flow regimes. Adapted from [10]. The boundaries between the different regimes are speculative. This work explores the onset of intermittent pathway flow.

However, many experimental observations show dynamic fluctuations in fluid interfaces. Intermittent flow pathways, whereby the flow pathways periodically disconnect and reconnect, have been observed during steady-state two-phase flow [8,9,10,11,12,13,14,15,16,17,18]. While the time-averaged saturation is constant, these intermittent pathways have a significant impact on fluid connectivity and, thus, the relative permeability of the fluids [19]. It has been observed that intermittent pathway flow is dependent on the competition between capillary and viscous forces, with the parameter space described by the capillary number and viscosity ratio, as highlighted in Figure 1 [8,10,13,15,19,20]. These dynamics have been observed to occur within the parameter space occupied by applications involving gas and water multiphase flow, such as carbon or hydrogen storage [4,21]. The destruction and creation of flow pathways will influence energy dissipation and the trapping of gas; thus, this will heavily influence the propagation of the gas plume in the subsurface [22,23].

While these fluctuations have been observed extensively in flow experiments, their relation to fluid and solid properties, as needed for predictive modelling, is still not well understood. This relates in particular to the location of those fluctuations; these dynamics predominantly occupy a small fraction of the pore space (with dynamics observed alongside connected pathway flow in some cases [15]), and they heavily influence fluid connectivity and the relative permeability of the fluid phases present. For example, Spurin et al. [23] observed fluctuations in the saturation of ±15% of the mean saturation, while the number of disconnected regions varied by ±70% of the mean value.

In this work, we explore the importance of the viscosity ratio as a parameter governing the onset of fluid interface reconfiguration. We systematically vary the viscosity ratio (see Figure 1 for the location of this work in the parameter space relative to previous work) and investigate how this variation impacts the frequency and locations of the interface fluctuations. Our setup allows for the direct visualisation of fluid reconfiguration via tomography in a carbonate sample. We determine the statistical signature associated with fluctuations under varying viscosity ratios, providing insights into the complex interplay between fluids and the porous medium.

## 2. Materials and Methods

### 2.1. Experimental Procedure

We conducted three experiments in a single cylindrical Estaillades carbonate sample, 5 mm in diameter and 20 mm in length. The sample was initially saturated with brine (de-ionised water doped with 15% wt. KI). Then, we simultaneously co-injected decane and brine, with the imaging of the sample occurring during a steady state, whereby the pressure drop measured across the sample had plateaued. For further details of the experimental design, see [9]. The fractional flow (the fraction of the total flow rate constituted by the brine flow rate) was kept constant at $f_{w}$ = 0.5, but the viscosity of the brine was increased by adding glycerol to the brine mixture. The viscosity ratio, M, is defined as

(1) $$M=\frac{\mu_{nw}}{\mu_{w}}$$

where the viscosity of the brine/glycerol was calculated using the models proposed in [24,25]. Note that the total flow rate was changed so that the capillary number was constant even with the change in viscosity, using the capillary number as defined in [10]. The flow rates and glycerol concentrations are listed in Table 1. The sample was not re-saturated with brine between changes in viscosity.

The sample was imaged at the TOMCAT beamline at the Swiss Light Source. It was exposed to filtered polychromatic X-ray radiation with a peak energy of about 26 keV. The filter was 2300 μm thick Silicon. An in-house developed GigaFRoST camera [26] and a high numerical aperture white-beam microscope (Optique Peter) with 4× magnification [27] were used, yielding an effective pixel size of 2.75 μm. Each tomogram contained 1000 projections over a 180° rotation. Each scan lasted 1 s, with 60 s between scans. With this temporal resolution, all interfaces were resolvable, i.e., no blurring was observed, as was the case in previous research [10,12,19].

**Figure 2:**
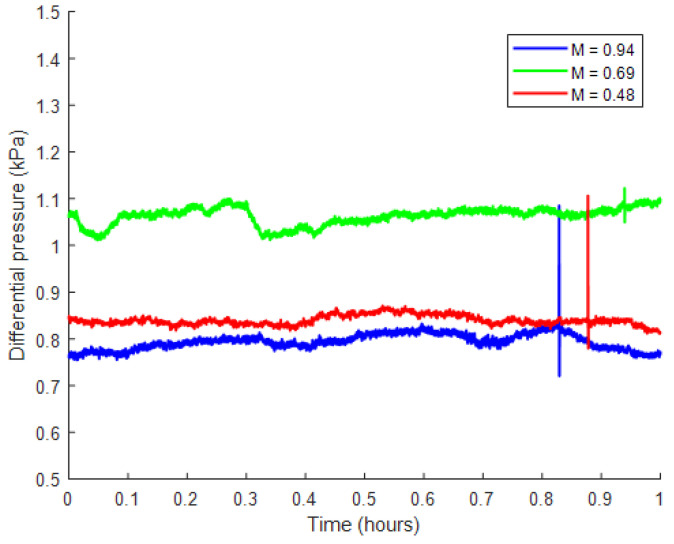
The pressure drop across the core during imaging for the three experiments. The pressure drop across the core was constant when averaged over time; this indicated that the experiment was in a steady state.

Images were taken during steady-state flow. This was determined by the pressure drop across the core plateauing. The pressure across the cores during flow is given in Figure 2.

### 2.2. Image Processing

**Figure 3:**
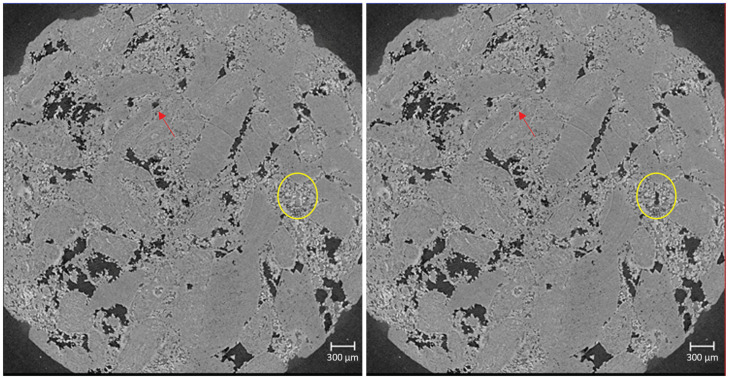
Raw image of the pore space (slice 746) for M = 0.48 at the timesteps 5 (**left**) and 45 (**right**). The NWP is the darkest phase, with changes in connectivity highlighted by the red arrow (imbibition event) and yellow circle (drainage event).

The images acquired have a voxel size of 2.75 μm. Each image analysed was 4224 × 4298 × 4263 μm in size, i.e., we imaged and analysed the middle 4.3 mm of a 21 mm long sample. The images were filtered with a nonlocal means filter to minimise noise while preserving the boundary between phases. The first image was taken with just de-ionised water in the pore space. This image was used to segment the pore space from the rock grains using a watershed segmentation algorithm. Then, the sample was saturated with the brine, and another image was taken; this is the brine-saturated image. All subsequent images with the decane and brine present were registered in 3D and then subtracted from the brine-saturated image; this results in a differential image whereby only the location of the nonwetting phase remains. From this, a simple greyscale value threshold can be used to segment out the nonwetting phase (NWP), i.e., the decane. The segmented pore space was overlain on this segmentation to locate the pore space occupied with brine. The full image processing workflow is described in [28]. We explore distinct clusters of the NWP, referred to as ganglia. An example of the pore-scale displacement processes is shown in Figure 3.

In this work, we explore the connectivity of the NWP, as it occupies the largest pores and is, therefore, less impacted by a finite image resolution.

### 2.3. Occupation Analysis

We analysed the segmented image sets with respect to the occupation of voxels with the NWP as a means to quantify the induced fluid flow fluctuations. The NWP saturation *S* was determined by

(2) $$S=\frac{N_{NWP}}{N_{NWP}+N_{WP}}$$

with the number of voxels assigned to the NWP, $N_{NWP}$, and wetting phase, $N_{WP}$. The average saturation, $S_{avg}$, subsequently becomes

(3) $$S_{avg}=\frac{1}{n_{t}}\sum_{t=1}^{n_{t}}S\left(t\right)$$

where $n_{t}$ denotes the number of timesteps. Although $S_{avg}$ and its temporal development provide an indicator for the steady state of the flow, it does not allow the quantification of the observed fluctuations. Therefore, we introduce a constant, maximum, and fluctuation NWP occupation, termed $\Omega_{s}$, $\Omega_{m}$, and $\Omega_{f}$, respectively. The constant NWP occupation $\Omega_{s}$ was determined by taking only those voxels into account which are assigned to the NWP in each image of a scan:

(4) $$\Omega_{s}=\frac{1}{N_{NWP}+N_{WP}}\sum_{n_{I}}\left(\overset{n_{t}}{\underset{t=1}{⋂}}I_{NWP}\right)$$

where the summation occurs over the voxels, $n_{I}$, in the data with $I_{NWP}=1$ if a voxel is filled with NWP and $I_{NWP}=0$ otherwise. Similarly, we find the maximum NWP occupation, $\Omega_{m}$, by taking all voxels into account, which are occupied at least once by NWP:

(5) $$\Omega_{m}=\frac{1}{N_{NWP}+N_{WP}}\sum_{n_{I}}\left(\overset{n_{t}}{\underset{t=1}{⋃}}I_{NWP}\right)$$

Finally, we compute the fluctuation occupation, $\Omega_{f}$, as the difference between the maximum and constant NWP occupation:

(6) $$\Omega_{f}=\Omega_{m}-\Omega_{s}$$

Additionally, we extracted the pore network of the complete pore space using the maximum ball method [29]. We then analysed the extracted pore networks with respect to the occupation of pores by using the NWP to characterise their dynamics throughout the experiment. Here, we assigned a pore to a phase depending on the voxel value at the centre of the inscribed sphere. Subsequently, we then analysed the changes in network connectivity and the sizes of the affected pores.

## 3. Results

### 3.1. The Effect of Viscosity on Fluid Dynamics

**Figure 4:**
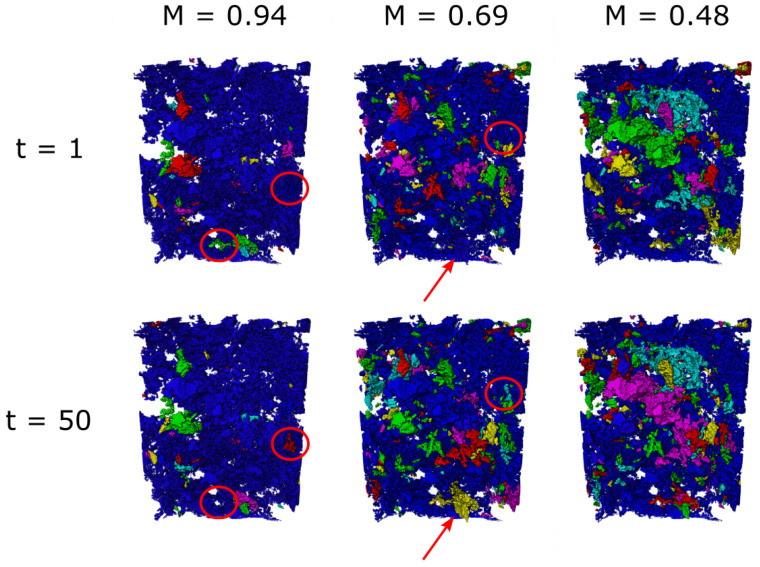
NWP ganglia distribution for the different viscosity ratios at two different time steps. Only the NWP is shown, with the brine and rock grains being transparent. Each distinct ganglion of NWP is given a different colour. A couple of connectivity changes are highlighted by the red circles and arrows.

For the highest viscosity ratio (M = 0.94), as shown in Figure 4, the NWP is predominately a single, continuously connected pathway, with only minor changes in saturation during a steady state. The red circles in Figure 4 show two regions where an otherwise disconnected ganglion connects to the main flow pathway across the core, highlighting that there were some changes in fluid connectivity during the experiment.

For the middle viscosity ratio (M = 0.69), there are more disconnected ganglia, with connection/disconnection events existing alongside a connected pathway across the core. For the lowest viscosity ratio (M = 0.48), the disconnected ganglia appear larger, with larger rearrangements also visible (shown by changing colours between the time steps in Figure 4). However, a connected pathway across the core is also present.

**Figure 5:**
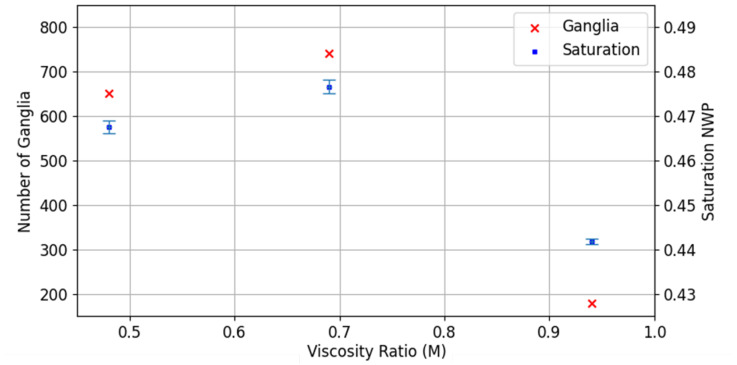
Number of Ganglia and averaged saturation with the NWP in dependency of the viscosity ratio *M*. The error bars indicate the standard deviation of the averaged saturation during imaging.

The saturation and number of NWP ganglia are shown in Figure 5. The competition between the viscous forces and capillary forces controls the invasion pattern of the NWP, with viscous fingers being more ramified than capillary fingers [30,31]. Thus, decreasing the viscosity in this context would decrease the saturation of the NWP. This is not the case for these experiments. We see a small increase in saturation for the lower viscosity ratio in Figure 5. This corresponds with an increase in the number of disconnected ganglia (Figure 5) and the volume of NWP fluctuating over the duration of the experiment. This suggests that the onset of intermittency plays a pivotal role in the fluid distribution and, therefore, the saturation.

Overall, differences in fluid distribution and connectivity are evident when the viscosity ratio is changed, with the onset of intermittent flow pathways seemingly controlling the fluid distributions across the core.

### 3.2. The Role of Fluctuations on Fluid Connectivity

**Figure 6:**
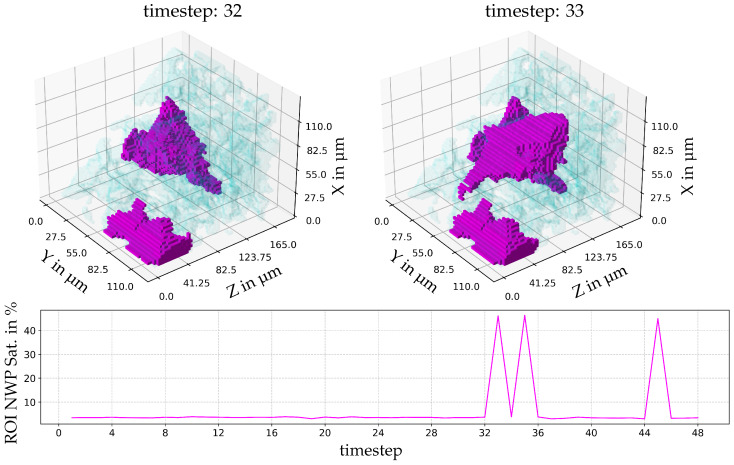
The largest event by volume and temporal development of the local temporal saturation changes for a viscosity ratio of M = 0.94. Below, the saturation of the pore space with NWP in the region of interest (ROI) is indicated. The wetting phase is indicated as blue, the NWP as magenta, and the rock grains are not shown. ROI in voxel indices xyz: [1300:1349][304:370][368:412].

In order to explore the impact of the different dynamics on the connectivity of the NWP as the viscosity ratio is altered, we explore the largest event by volume for each viscosity ratio. Figure 6 shows the largest event for M = 0.94. Here, the NWP interface oscillates (the difference between time steps 32 and 33 is shown). This interface oscillation occurs three times, with no obvious periodicity. The event does not change the connectivity of the NWP.

**Figure 7:**
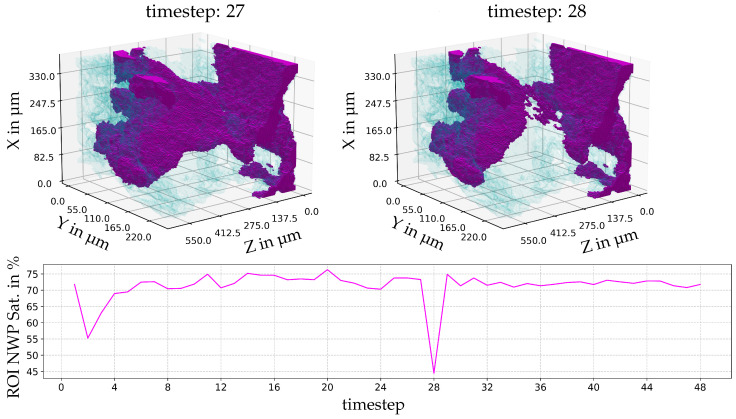
The largest event by volume and temporal development of the local temporal saturation changes for a viscosity ratio of M = 0.69. Below, the saturation of the pore space with NWP in the ROI is indicated. The wetting phase is indicated as blue, and the NWP is magenta. ROI in voxel indices xyz: [1172:1310][855:945][496:716].

For the intermediate viscosity ratio (M = 0.69), Figure 7, the largest event involves the connection between neighbouring ganglia. This connect event occurs twice and also disconnects twice.

**Figure 8:**
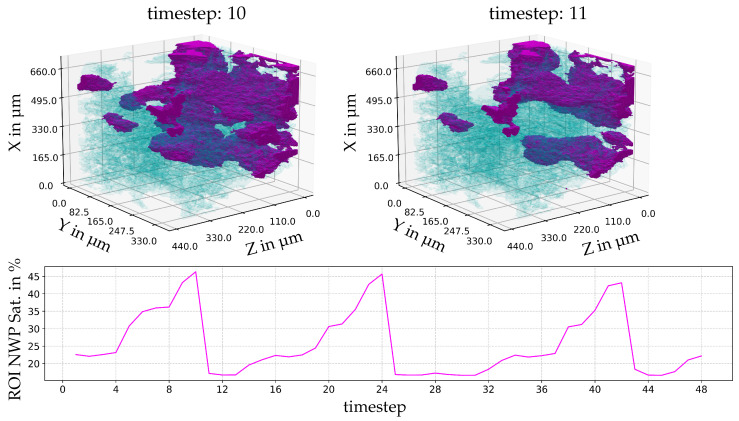
The largest event by volume and temporal development of the local temporal saturation changes for a viscosity ratio of M = 0.48. Below, the saturation of the pore space with NWP in the ROI is indicated. The wetting phase is indicated as blue, and the NWP is magenta. ROI in voxel indices xyz: [1153:1413][748:877][644:801].

For the lowest viscosity ratio (M = 0.48), Figure 8, the largest event connects a region that would otherwise be completely disconnected from flow. This event occurs three times during scanning; it has a more noticeable periodicity. The NWP saturation increases slowly with time, but the water invasion occurs rapidly, which is expected in a water-wet porous medium.

For the highest viscosity ratio, we have no significant intermittent pathway flow. The onset of dynamics begins for the intermediate viscosity ratio and develops further for the lowest viscosity ratio. Initially, the dynamics are small and do not influence the large-scale connectivity of the NWP. With the onset of intermittency, we observed that the connections become important in controlling the connectivity of the NWP. The connections become more sustained for the lowest viscosity ratio, with large-scale rearrangement of fluid connectivity.

### 3.3. The Evolution of Fluctuations with Viscosity Ratio

**Figure 9:**
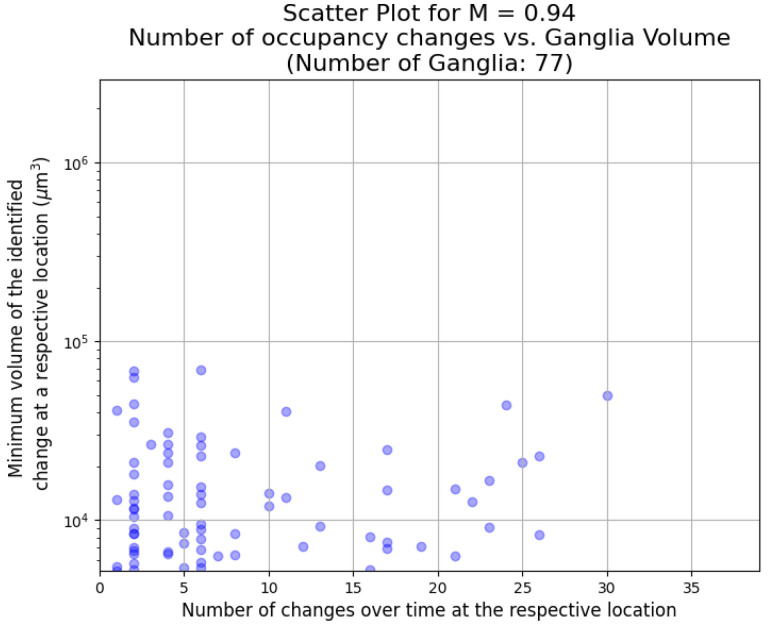
The number of occupancy changes for ganglia greater than 250 voxels cubed plotted against ganglion size for M = 0.94. The largest event during this experiment is shown in Figure 6.

**Figure 10:**
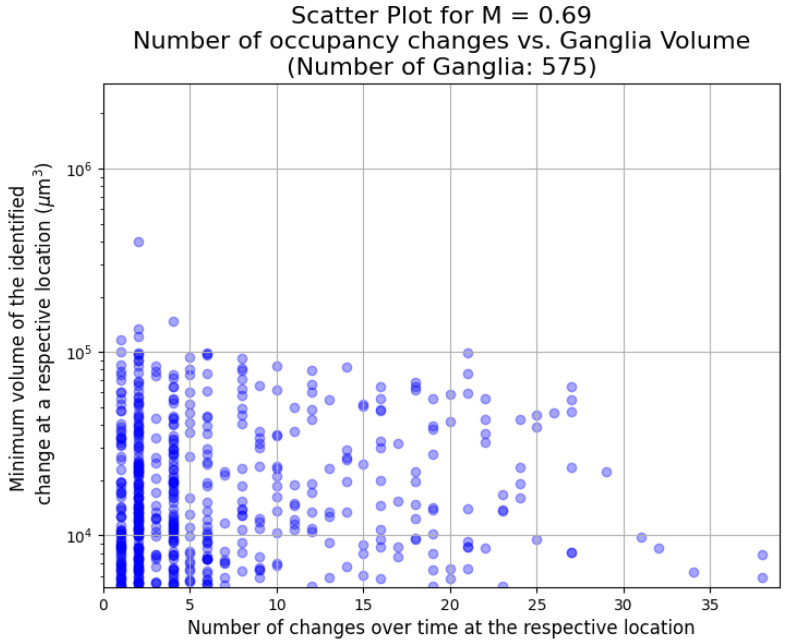
The number of occupancy changes for ganglia greater than 250 voxels cubed plotted against ganglion size for M = 0.69. The largest event during this experiment is shown in Figure 7.

**Figure 11:**
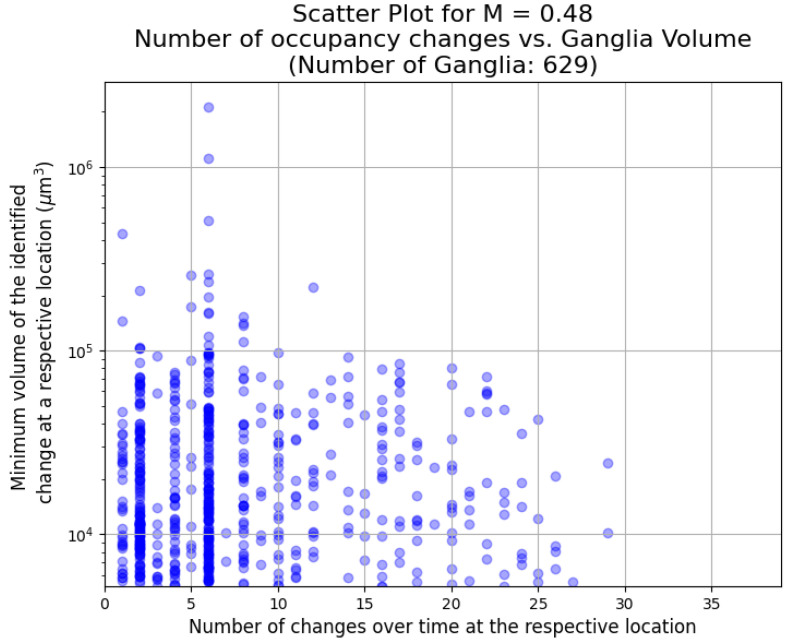
The number of occupancy changes for ganglia greater than 250 voxels cubed plotted against ganglion size for M = 0.48. The largest event during this experiment is shown in Figure 8.

We examine the evolution of the dynamics as the viscosity ratio by observing the changes in the size and frequency of the events. This is shown in Figure 9, Figure 10 and Figure 11 for a viscosity ratio of M = 0.94, M = 0.69, and M = 0.48, respectively. For the highest viscosity ratio (M = 0.94), the events are smaller, and there are fewer events. The frequency range is similar to the lower viscosity ratios. For the lower viscosity ratios, there are larger events over all frequencies. In all cases, there are more smaller events than larger events (the same observations were made in [32]). However, one needs to note that the smaller an event, the more it may be affected by image noise and artefacts.

**Figure 12:**
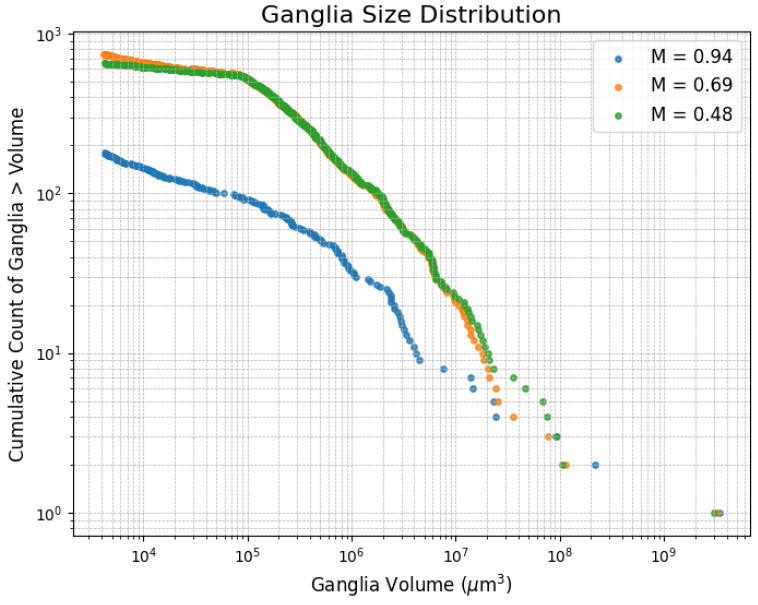
Ganglia size distribution for the experiments. Cumulative count of ganglia greater than a given volume.

The ganglia size distribution is shown in Figure 12. Here, we observe a noticeable shift from the highest viscosity ratio to the other two viscosity ratios. For the middle viscosity ratio, there are more larger ganglia and fewer smaller ones.

### 3.4. Impact of Fluctuations on the NWP Pore Network

**Figure 13:**
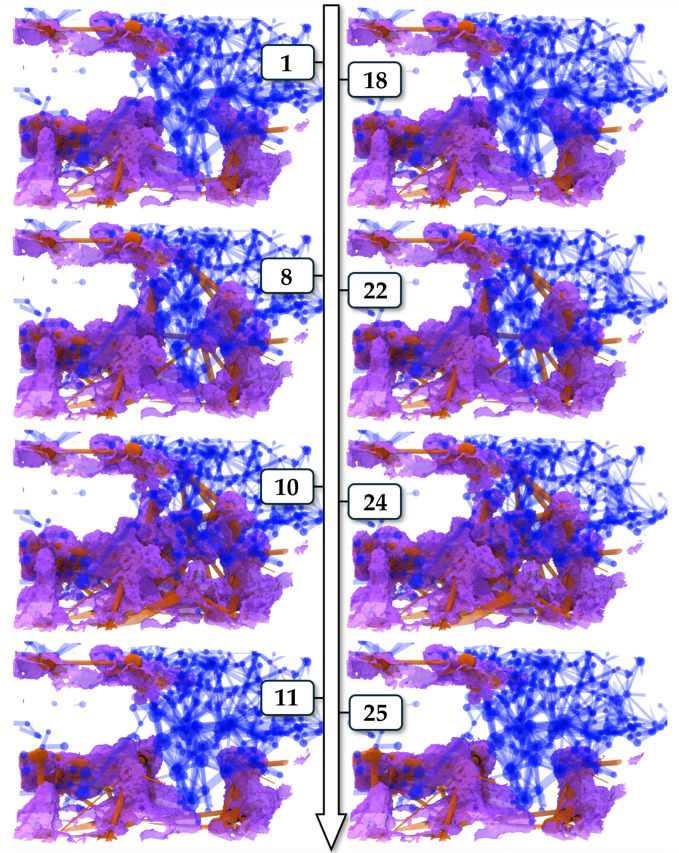
Snapshots of oscillating fluid fluctuations for M = 0.48, with the filling status of the extracted pore network. The NWP is indicated by the violet contour, the blue network parts are purely filled with the wetting phase, and the red parts are filled by the NWP. The numbers indicate the scan within the time series.

We assessed the impact of the intermittent behaviour on the NWP network by extracting a pore network (Appendix B) of the solid and identifying fluid occupancy in the individual pore bodies and throats at each time step. An example of the oscillating nature of the local fluid fluctuation and its impact on the connectivity of the pathway is displayed in Figure 13 for the viscosity ratio M = 0.48. There, the pores of the extracted network are coloured with respect to their associated phase, and only the throats assigned to the pores of the same phase are shown.

With the progress of the experiment, the interface develops, and a slow increase in the local occupancy of the NWP can be observed in time steps 1 to 10. In contrast, a sudden displacement of the NWP by the wetting phase is visible between steps 10 and 11. This behaviour appears again in time steps 18 to 25, with almost identical fluid configurations, indicating that the nature of those oscillations is rather deterministic and not a random event.

Additionally, the change in connectivity in the pore network is clearly visible. However, it can also be observed that not all indicated connections between pores are actual connections between the associated phases.

**Figure 14:**
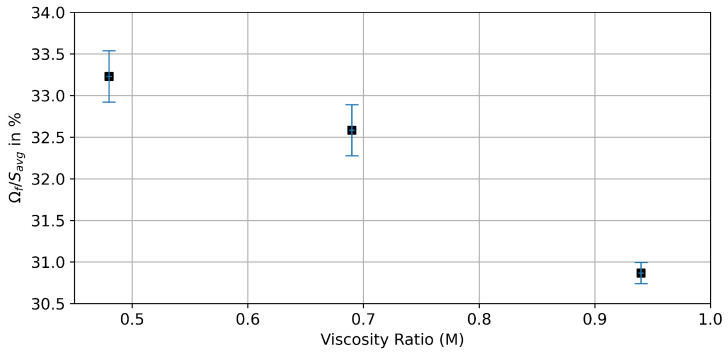
Development of the fluctuating domain $\Omega_{f}$ compared to the average saturation $S_{avg}$ in dependency of the viscosity ratio *M*. The error bars indicate the standard deviation of $S_{avg}$.

The statistical evaluation of those fluctuations is shown in Figure 14, where the dependency of the domain fraction affected by the fluctuations $\Omega_{f}$ on the viscosity ratio *M* is displayed. There, it can be seen that a domain of 30–34% of the average saturation is affected by those fluctuations. However, the NWP saturation of the complete core is comparatively constant over the time series with a standard deviation of <1%.

**Figure 15:**
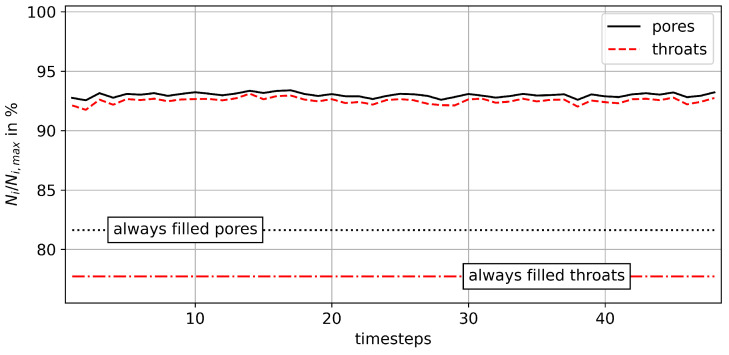
Progression of the amount of pores filled with the NWP in the case of M = 0.69. The limit at 100% is determined by accounting for all pores that are filled with the NWP during at least one timestep. Note that the affected pores and throats of the extracted network represent below 5% of the total network volume. The results for M = 0.48 and M = 0.94 are provided in Figure A1 and Figure A2, respectively.

Furthermore, the relative number densities of the pores and throats in the extracted pore network are shown for the example of *M* = 0.69 in Figure 15. Similar to the development of the saturation inside the sample, the total number of pores and throats occupied by NWP is comparatively constant. Yet again, the actually filled pores and throats vary significantly throughout the experiment. Note that the affected volume of the extracted pores accounts for less than 5% of the complete pore network. This is caused by the filtering method described in Section 2.3, which requires that the centre of a pore coincides with the NWP. Therefore, it is necessary to consider pore filling at high local saturation levels, and subsequently, smaller pores are more likely to be affected.

With respect to those results, it can be concluded that a smaller viscosity ratio increases the intensity of local fluctuations. Additionally, those fluctuations are confined to a local redistribution of liquids, whereas the complete pore space is barely affected.

## 4. Conclusions

In this work, we have shown that the viscosity ratio between the fluids present impacts the flow dynamics during two-phase flow in a porous medium beyond the effect of the capillary number. The fluctuations cause significant changes in connectivity that cannot be discounted when considering the transport and trapping of the fluids.

We have demonstrated the differences in ganglion size and distribution caused by altering the viscosity ratio. The dynamics are complex and nonlocal; this means that finding the key dynamics that are representative of the sample can be arduous. By exploring the statistical signature of the ganglion, we were able to deduce the changes caused by changes in viscosity. However, a workflow to automate this discovery would allow for quicker and more objective studies in the future. Future work will include incorporating these findings into relative permeability calculations.

## Figures and Tables

**Table 1:** List of decane/brine experiments conducted at the synchrotron.

Viscosity Ratio	Total Flow Rate (mL/min)	Glycerol: Brine	Capillary Number
0.94	0.1	0	1.6 $\times \;10^{-6}$
0.69	0.084	1:9	1.6 $\times \;10^{-6}$
0.48	0.075	2:8	1.7 $\times \;10^{-6}$

**Figure A1:**
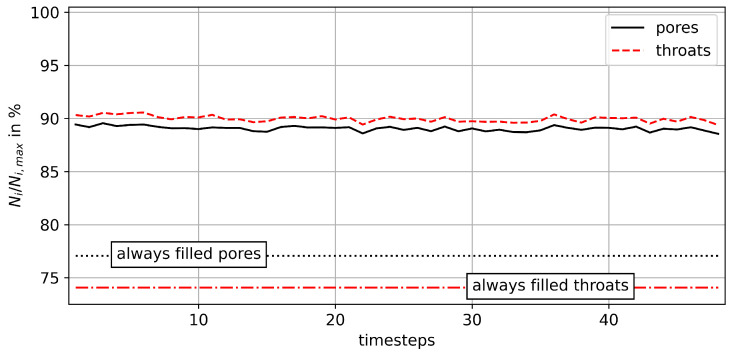
Development of relative amount of filled pores and throats in the case of M = 0.48.

**Figure A2:**
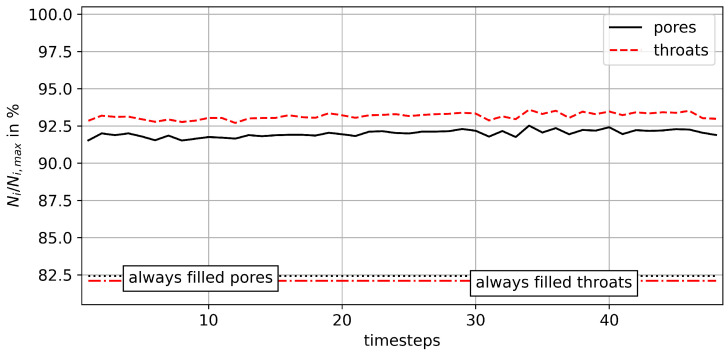
Development of relative amount of filled pores and throats in the case of M = 0.98.

**Figure A3:**
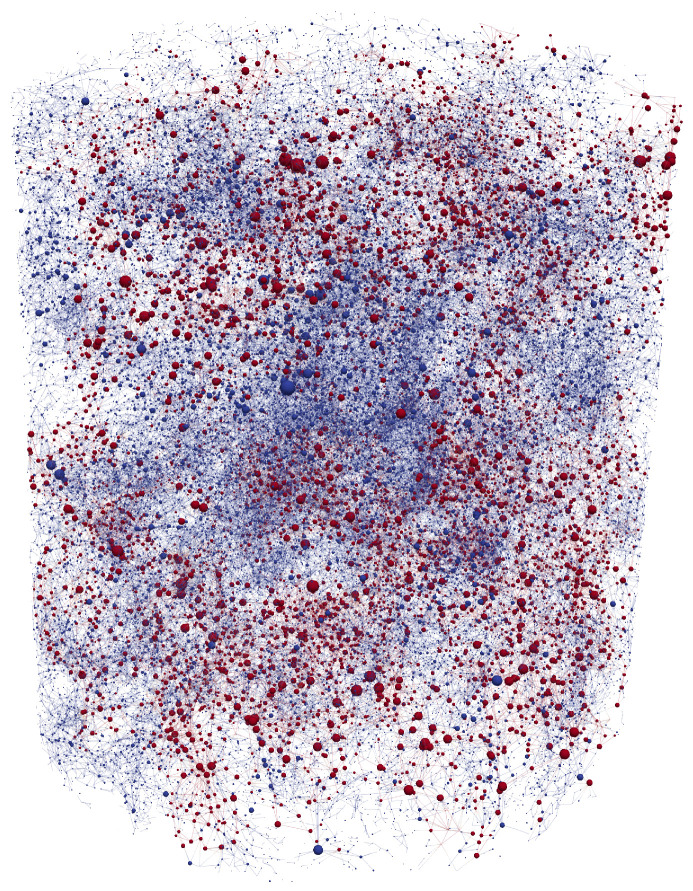
Pore network extracted for M = 0.98 at the first timestep. Blue denotes the wetting phase, and red denotes the nonwetting phase.

## Data Availability

The full dataset is available at https://doi.org/10.4121/ba6e70f8-affa-4c60-82f0-24bff9052676.

## Abbreviations

$\Omega_{f}$	relative occupied volume of fluctuations
$\Omega_{m}$	maximal relative occupied volume
$\Omega_{s}$	minimal relative occupied volume
*I*	value of discrete voxel in scan
M	viscosity ratio
*S*	saturation
$S_{avg}$	average saturation of time series
WP	wetting phase
NWP	non wetting phase
ROI	region of interest
